# Mechanism of action of lenalidomide in hematological malignancies

**DOI:** 10.1186/1756-8722-2-36

**Published:** 2009-08-12

**Authors:** Venumadhav Kotla, Swati Goel, Sangeeta Nischal, Christoph Heuck, Kumar Vivek, Bhaskar Das, Amit Verma

**Affiliations:** 1 Department of Medicine, Albert Einstein College of Medicine, Bronx, USA; 2 Harrison Department of Surgical Research, University of Pennsylvania, Philadelphia, USA; 3 Developmental and Molecular Biology, Albert Einstein College of Medicine, Bronx, USA

## Abstract

Immunomodulatory drugs lenalidomide and pomalidomide are synthetic compounds derived by modifying the chemical structure of thalidomide to improve its potency and reduce its side effects. Lenalidomide is a 4-amino-glutamyl analogue of thalidomide that lacks the neurologic side effects of sedation and neuropathy and has emerged as a drug with activity against various hematological and solid malignancies. It is approved by FDA for clinical use in myelodysplastic syndromes with deletion of chromosome 5q and multiple myeloma. Lenalidomide has been shown to be an immunomodulator, affecting both cellular and humoral limbs of the immune system. It has also been shown to have anti-angiogenic properties. Newer studies demonstrate its effects on signal transduction that can partly explain its selective efficacy in subsets of MDS. Even though the exact molecular targets of lenalidomide are not well known, its activity across a spectrum of neoplastic conditions highlights the possibility of multiple target sites of action.

## Thalidomide is the first immunomodulatory drug with multiple effects on the immune system

**Figure 1:**
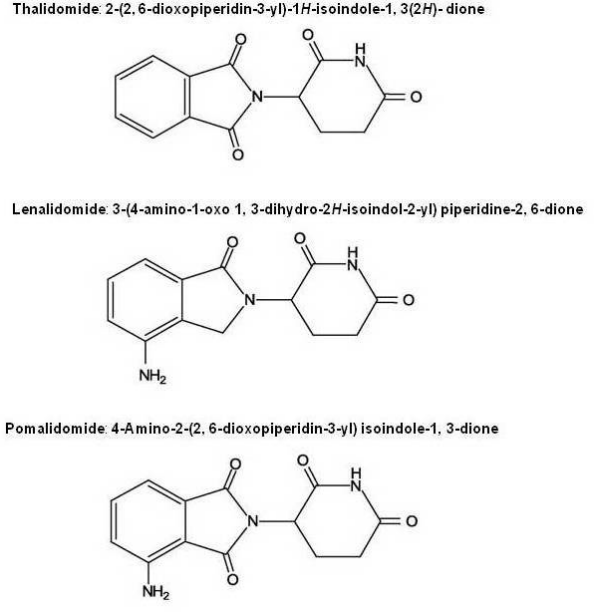
**Chemical structures of thalidomide, lenalidomide and pomalidomide**.

Immunomodulatory drugs (IMiDs) CC-5013 (Revlimid TM, Lenalidomide) and CC-4047 (ActimidTM, Pomalidomide) are a series of synthetic compounds derived using structural modifications of the chemical structure of thalidomide. Thalidomide (a-(N-phthalimido) glutaramide) was synthesized in Germany, in 1954, from glutamic acid, to be used as a sedative and hypnotic anti-emetic drug, indicated to treat morning sickness in the first trimester of gestation. Thalidomide was banned in the 1960s because of the reports of congenital malformations like phocomelia associated with its use in pregnant women. One of the possible hypothesis to explain this teratogenecity is that thalidomide creates oxidative stress by with subsequent downregulation of Wnt and Akt survival pathways which induces apoptosis during early embryonic limb development resulting in limb truncations[1]. Following an observation in 1965 that thalidomide administration improved the inflammatory lesions of erythema nodosum leprosum (ENL) in a patient suffering from sleep difficulty, the use of thalidomide continued. Eventually in 1998, FDA approved the drug for the treatment of ENL, with tight restrictions on its marketing. ENL is an immune complex mediated inflammatory reaction that occurs during therapy in lepromatous leprosy patients. It is commonly associated with systemic symptoms, and constitutes a medical emergency with urgent need of therapy with anti-inflammatory/immunomodulatory drugs to prevent long term disabilities. Research into the mechanism of action of thalidomide unraveled an immunological and immunomodulatory basis for the effect, notably inhibition of denovo IgM antibody synthesis[2] by possibly affecting the macrophages, B-cells, helper or suppressor lymphocytes, decreasing TNF-α synthesis and modulating the T cell subsets by increasing the T-helper population after therapy[3]. TNF-α is a potent pro inflammatory cytokine, and is also involved in the pathogenesis of neural damage in leprosy. The inhibitory effect of thalidomide on TNF-α is a consequence of increased degradation of its mRNA due to the drug [4]. Thalidomide also regulates the levels of IL-6 and IFN-γ in ENL patients, further contributing to the immunomodulatory mechanism of action. Interest in thalidomide as a neoplastic agent intensified after the demonstration of antiangiogenic activity in animal models. The recognition that angiogenesis plays an important pathogenic role in multiple myeloma as reflected by increased bone marrow microvascular density and VEGF (vascular endothelial growth factor) levels, prompted the clinical use of thalidomide in relapsed/refractory multiple myeloma. With the recognition of adverse effects like neuropathy, deep vein thrombosis, and sedation, more potent and safer analogues were developed by Celgene. Lenalidomide is one such analogue which has been extensively tested and proven to be more potent than thalidomide and has fewer adverse effects compared to thalidomide. Another newer thalidomide analogue is pomalidomide. Figure 1 consists of the chemical structures and names of these three compounds and Table 1 illustrates the differences amongst them.

**Table 1:** Differences between thalidomide, lenalidomide and pomalidomide

**Name**	**Thalidomide**	**Lenalidomide**	**Pomalidomide**
**Empirical Formula**	C_13_H_10_N_2_O_4_	C_13_H_13_N_3_O_3_	C_13_H_11_N_3_O_4_
**Molecular weight**	258.2	259.3	273.2
**Chemical Structural**	Thalidomide has two oxo groups in Phthaloyl ring	Lenalidomide has amino group at 4th position and single oxo group in Phthaloyl ring	Pomalidomide has amino group at 4th position and two oxo groups in Phthaloyl ring
**Effects on T-cell proliferation**	Thalidomide stimulates T cell proliferation and increases IFN-γ and IL-2 production	Lenalidomide is 100–1000 times more potent in stimulating T cell proliferation and IFN-γ and IL-2 production than thalidomide	Pomalidomide is similar to lenalidomide, in addition, it also enhances transcription factor T-bet, which reverts Th2 cells into Th1 like effector cells in vitro

**Adverse Effects**	Thalidomide has higher incidence of side effects like sedation, neuropathy and constipation.	Lenalidomide has lower incidence of adverse effects namely sedation, constipation and neuropathy than thalidomide.	Pomalidomide has lower incidence of adverse effects like sedation, constipation and neuropathy than thalidomide.

**Teratogenecity**	Thalidomide is a known teratogen.	Lenalidomide is not teratogenic in rabbit models	Pomalidomide is a known teratogen.

## Mechanism of action of Lenalidomide

The clinical evidence for therapeutic potential of lenalidomide in various malignant conditions is consistent with the multitude of pharmacodynamic effects that have been shown in vitro and in animal models. Studies have shown that lenalidomide may work through various mechanisms in different hematologic malignancies. These mechanism involved direct cytotoxicity as well as through indirect effects on tumor immunity. Thus the differential efficacy noted with lenalidomide therapy among various disease states can possibly be explained individual's immune status and disease specific pathophysiology. Following are the different mechanisms explained by which lenalidomide acts in the body.

## Immunomodulation

The immune system is comprised of cellular (macrophages, dendritic cells, NK cells, T cells and B cells), and humoral components (antibodies, cytokines). The immune system can prevent development of cancers by eliminating or suppressing oncogenic viral infections, altering the inflammatory milieu conducive to tumor genesis, and by immune surveillance by identifying and destroying transformed cells before they can cause harm[5].

Lenalidomide has been shown to modulate different components of the immune system by altering cytokine production, regulating T cell co stimulation and augmenting the NK cell cytotoxicity. Immunomodulatory properties of Lenalidomide are implicated in its clinical efficacy in multiple myeloma, CLL and myelodysplastic syndromes; where the disease pathogenesis involves in part a deregulated immune system in the form of altered cytokine networks in tumor microenvironment, defective T cell regulation of host-tumor immune interactions, and diminished NK cell activity.

### Altering cytokine production

Cytokines are soluble proteins secreted by hematopoietic and non hematopoietic cell types and are critical for both innate and adaptive immune responses. The expression of cytokines by cells may be altered in immunological, inflammatory, infectious and neoplastic disease states. Cytokines in turn exert their effects by influencing gene activation, growth, differentiation, functional cell surface molecule expression and cellular effector function. A coordinated cellular and humoral (cytokines, antibodies) interactions facilitate tumor destruction.

Lenalidomide has been shown to inhibit production of pro inflammatory cytokines TNF-α, IL-1, IL-6, IL-12 and elevate the production of anti-inflammatory cytokine IL-10 from human PBMCs[6]. The downregulation of TNF-α secretion is particularly striking and is up to 50,000 times more when compared to thalidomide[7]. TNF-α is a highly pleiotropic cytokine produced primarily by monocytes and macrophages and plays an important role in protective immune responses against bacterial and viral infections. Elevated TNF-α production is implicated in the pathogenesis of various hematologic malignancies and may be partly responsible for stem cell apoptosis and ineffective hematopoiesis seen in MDS [8]. TNF-α levels in CLL patients are also elevated and exhibit a significant decrease as early as 7 days after lenalidomide treatment. These reductions correlate with cytoreduction suggesting a casual relationship with tumor growth [9].

Similarly, reduction in IL-6 and TNF-α levels could explain the action of lenalidomide in multiple myeloma. IL-6 inhibits the apoptosis of malignant myeloma cells and helps in their proliferation[10]. Lenalidomide downregulates the production of IL-6 directly and also by inhibiting multiple myeloma (MM) cells and bone marrow stromal cells (BMSC) interaction [11,12], which augments the apoptosis of myeloma cells[13]. The precise mechanism of TNF-α downregulation by lenalidomide is not known, however thalidomide has been shown to increase the degradation of TNF-α mRNA [4,14]. It is possible that lenalidomide may work through similar mechanisms.

### T cell activation

**Figure 2:**
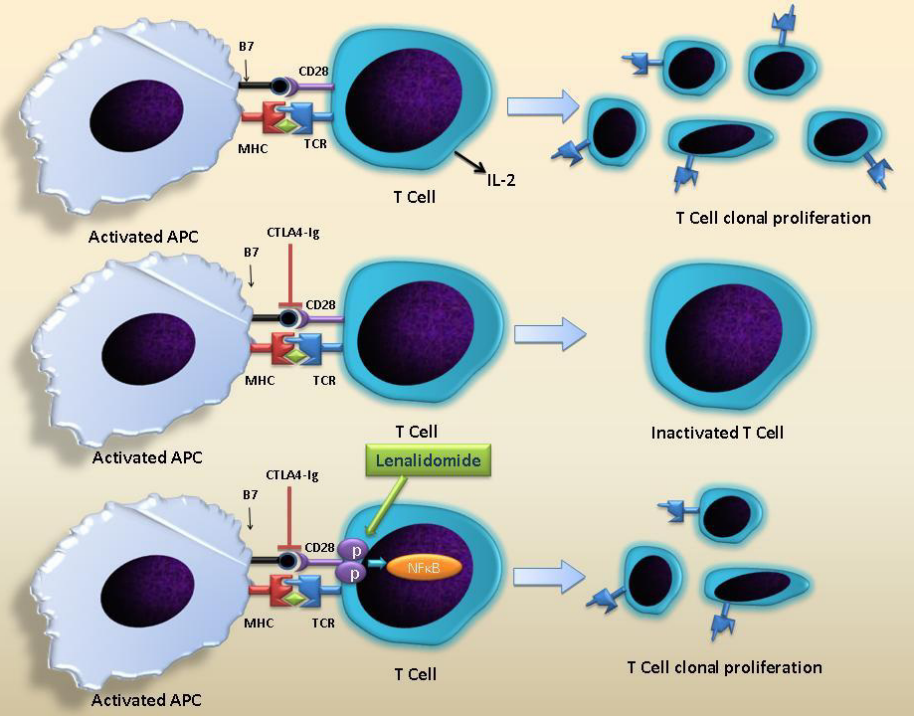
**T cell activation**. B7-CD28 co-stimulation pathway is needed for T cell activation and CTLA4 Ig blocks this pathway leading to T cell inactivation. Lenalidomide acts by directly inducing tyrosine phosphorylation of CD28 on T cells leading to activation of downstream targets such as PI3K, GRB-2-OS, and NF-κb, thus partially overcoming CTLA4 Ig blockade and leading to T cell clonal proliferation.

T cells are important effectors of immune response and their activation is tightly regulated to prevent auto reactivity. T cell activation involves the presentation of the peptide fragments displayed by antigen presenting cells (APCs) to the T cell receptor (TCR) and it is this interaction that gives specificity to the response. However this interaction alone is not sufficient if a T cell has to generate an effective response against the antigen. A secondary interaction of B7 molecule on APC and CD28 on the T cell surface provides the co stimulatory signal that augments the T cell response and aids in T cell proliferation, differentiation, and survival followed by a cascade of cytokine and cellular responses[15].(Figure 2). IMiDs, including lenalidomide act on T cells via B7-CD28 co stimulatory pathway. Blockade of this interaction using the CTLA-4-Ig, B7 blocking antibody, is partially overcome by IMiDs. IMiDs do not up regulate expression of CD28 and B7 on T cells and APCs respectively but they can directly induce tyrosine phosphorylation of CD28 on T cells leading to activation of downstream targets such as PI3K, GRB-2-OS, and NF-κb. This might explain their ability to partially overcome CTLA4 Ig blockade[16]. T cell co-stimulation by lenalidomide leads to an increased Th1 type cytokine response resulting in increased secretion of IFN-γ and IL-2 that in turn stimulate clonal T cell proliferation and NK cell activity[6,17].

IMiDs have been shown to stimulate both cytotoxic CD8+ as well as helper CD4+ cells[18]. Their effects on T helper cells can potentially mediate Th1 type antitumor immunity in response to tumor cell vaccination in animal models[17]. The IMiD, CC-4047 (pomalidomide) enhanced partially protective antitumor effect of whole tumor cell vaccination in mice and generated long term immunity against subsequent live tumor challenge[17]. In vivo production of IFN-γ correlated with the tumor protection. When nude mice lacking T cells were exposed to IMiD and tumor cells during the priming phase, they did not demonstrate protection from the tumor, demonstrating that T cells are needed for tumor immunity. The IMiD drug itself was shown to have no direct anti tumor effect on growth inhibition or expression of co stimulatory molecules, ruling out direct cytotoxic effects. These effects can also partly explain the beneficial effects of lenalidomide in MDS. Clonal expansion of abnormal hematopoietic suppressive T cells are believed to have a pathogenic role in ineffective erythropoiesis of patients with MDS and 50% of the patients with MDS were shown to have clonal T cells compared to 5% in age matched controls[19]. It is possible that lenalidomide may affect certain T cell subsets and result in hematologic improvements in MDS patients.

### Augmentation of NK cell function

Natural Killer (NK) Cells comprise 2% of the circulating lymphocytes and are an important component of innate immunity. NK cells are not driven by specificity to antigens unlike T cells or B cells and are able to respond rapidly on contact with the target cell (cancer, viral infected) and kill the cell with antibody dependent cell mediated cytotoxicity(ADCC) and natural cytotoxicity. Natural killer cells also contribute to immunoregulation by secreting cytokines like IFN-γ and TNF-α. Modulation of NK cell function is also believed to contribute to the anti tumor activity of Lenalidomide in MDS, MM and CLL.

Davies et al examined the potential immunomodulatory effects of thalidomide and its analogues in patients with multiple myeloma. The in vitro/in vivo role of NK cell cytotoxicity of MM cells in thalidomide treated patient was supported by the observation that the cell killing was not MHC restricted and CD56(NK cell) depletion in vitro inhibited killing of drug treated multiple myeloma cells[20]. Furthermore, treatment with Thalidomide was also accompanied by increased NK cell numbers and IL-2 levels. The precise mechanism whereby IMIDS increase the NK cell number or augment its cytotoxicity is not well known and it is possible that these effects may be indirect. Hayashi et al in their study of IMiDs in MM cell lines have demonstrated that when culturing PBMC with IMiDs leads to 1.2–1.3 fold increase in the percentage of CD56 cells. IMiDs enhanced ADCC when 51 Cr-labelled MM cells that express CD40 were incubated with rhuCD40 and then subsequently treated with PBMC cells incubated in the presence of IMiDs for 5 days. The increase in NK cell function may be related to the increase in IL-2 production by the T cells as the presence of a monoclonal Ab against IL-2 R blocked the NK cell cytotoxicity. IMiDs also were shown not to directly activate the NK cells, as evidenced by lack of phosphorylation of signaling molecules (ERK/p38MAPK/Akt/PKC) in NK cells[21].

Lenalidomide also enhanced the NK cell mediated ADCC in a series of functional in vitro studies using Rituximab coated NHL cell lines, Trastuzumab coated breast cancer cells expressing Her2 and cetuximab coated colon cancer cells positive for EGFR expression. The cell killing was increased in a dose dependent manner and presence of IL-2 was required to achieve cell killing[22]. In another study [23], IFN-γ production by NK cell in rituximab coated NHL cell lines pretreated with lenalidomide, was induced with the interaction of Ig G with FC-γ receptors in the presence of IL-2 or IL-12. Thus, lenalidomide enhanced Fc-γ receptor signaling may also play a role in increasing the potency of NK cells.

## Anti-angiogenesis activity

The growth of the primary and metastatic tumors requires the development of new blood vessels, a process described as angiogenesis. Tumors possess the ability to promote the formation of new blood vessels from preexisting host capillaries at a critical phase of the tumor development when the balance of pro- angiogenic and anti-angiogenic factors is altered. Vascular endothelial growth factor (VEGF) and its receptors are required for the formation of blood vessels during embryonic development, wound healing, and carcinogenesis. Tumors are more dependent on the VEGF-Receptor signaling for growth and survival compared to normal endothelial cells [24]. Early studies showed that Thalidomide had anti angiogenic activity in a rabbit model of corneal neovascularization that was induced as a response to bFGF[25]. This report led to its use in Multiple Myeloma, where it demonstrated clinical benefit and was approved for use by the FDA. Thalidomide and the newer IMiDs have also been shown to significantly decrease the expression of angiogenic factors VEGF and Interleukin-6 (IL-6) in multiple myeloma; thereby reducing angiogenesis and hence contributing to clinical activity in multiple myeloma[26]. The newer IMiDs were found to be 2–3 times more potent compared to thalidomide in antiangiogenic activity in various vivo assays [27] The antiangiogenic activity of both thalidomide and IMiDs has also been shown to be independent of immunomodulatory effects[28].

VEGF receptors are overexpressed on blast cells in dysplastic marrows in MDS patients [29]. Increased plasma levels of VEGF R have also been correlated with lower remission rate in patients with myelodysplastic syndromes. A recent study in 35 MDS patients with del 5 q showed a marked decrease in bone marrow vascularity subsequent to lenalidomide therapy. This reduction in vascularity correlated with clinical responses. However VEGF levels and VEGFR levels did not change significantly even though vascularization was decreased, supporting the notion that lenalidomide may uncouple angiogenesis from the effect of VEGF[30]. Apart from alteration in the levels of VEGF, analysis of signal transduction events show that lenalidomide partially inhibits Akt phosphorylation after VEGF stimulation in endothelial cells and also has inhibitory effects on phosphorylation of Gab1, a protein upstream of Akt 1[31,32]. These observations demonstrate that IMiDs may affect angiogenesis by multiple mechanisms.

## Direct anti tumor activity

Lenalidomide treatment has also shown anti proliferative activity against MDS and MM cells in the absence of immune effector cells[33]. Malignant plasma cells derived from refractory cases of myeloma were shown to be susceptible to IMiD induced growth arrest. Lenalidomide has also been shown to inhibit proliferation in Burkitt's Lymphoma cell lines by causing dose dependant cell cycle arrest in G0-G1 phase[34]. Lenalidomide upregulated Cyclin dependant kinase (CDK) Inhibitor, p21 waf-1, a key cell cycle regulator that modulates the activity of CDKs. Similar reductions in CDK2 activity have been demonstrated in myeloma derived cell lines, U266 and LP-1[34]. In contrast, the normal B cells obtained from healthy donors were immune from growth inhibition and did not show any upregulation of p21 expression after 3 days of lenalidomide treatment. In other studies, thalidomide and its analogues have also been shown to induce apoptosis in MM cell lines[35]. Effects on apoptosis in MM cells is secondary to increased potentiation of TNF related Apoptosis inducing ligand (TRAIL), inhibition of apoptosis protein-2, increased sensitivity to Fas mediated cell death, and up regulation of caspase-8 activation, down regulation of caspase-8 inhibitors (FLIP, cIAP2), down regulation of NF-κb activity and inhibition of prosurvival effects of IGF-1[36]. The proapoptotic activity of IMiDs has also been demonstrated in CLL. Lenalidomide was shown to induce apoptosis and affect the Phosphotidylinositol pathway in CLL cells by decreasing activation of pro-survival kinases, erk1/2 and Akt2[37].

Interestingly, lenalidomide has shown opposite effects on the growth of normal progenitors. When cord derived CD34+ progenitors cells were cultured in expansion medium supplemented with lenalidomide, there was a dose dependent increase in the total number of CD34 cells after 6 days of culture [34]. p21 was upregulated in normal Cd34 cells, but did not affect the CDK2 activity in contrast to Nawalma cells (Burkitt's lymphoma cells).

While the transfusion independence seen with lenalidomide use in MDS can be explained by the normal progenitor expansion, the dose dependent cytopenias that are common with early treatment cycles of lenalidomide may be a result of inhibition of proliferation of abnormal clonal cell populations in the marrow.

## Effects on multiple myeloma microenvironment

Lenalidomide exerts its distinct anti myeloma effects by altering the myeloma microenvironment. In multiple myeloma, osteoclasts lead to bone resorption and secrete survival factors for MM cells. The interaction between MM cells and BMSC in turn leads to increased production of IL-6 and other growth factors for MM cells and osteoclasts[38]. Lenalidomide directly decreases the formation of tartrate- resistant acid phosphatase(TRAP)- positive cells which form osteoclasts [11]. Additionally, it decreases αVβ3-integrin levels, an adhesion molecule needed for osteoclast activation and downregulates cathepsin K, a major cysteine protease expressed in osteoclasts, pertinent for matrix degradation in the resorption process[11]. It downregulates the important mediators of osteoclastogenesis such as transcription factor PU.1 and MAP kinase pERK and reduces the levels of bone remodeling factor -receptor activator of nuclear factor-kappaB ligand. Immunomodulators are also known to decrease the cell surface adhesion molecules such as ICAM-1, VCAM-1 and E -selectin [12] and inhibit the adhesion of MM cells to BMSC. Thus, lenalidomide interferes with the synergism amongst the osteoclasts, MM cells and BMSC and decreases osteoclastogenesis by acting at various levels.

## Selective efficacy in cells with deletion of chromosome 5q

The del 5q syndrome is now recognized as a distinct pathologic subtype of MDS with markedly better clinical responses with lenalidomide treatment compared to non del 5q MDS patients. The exact mechanism of action of lenalidomide on del 5q clones is not known, but there appears to be several candidate genes (tumor suppressor) whose expression may be modulated by lenalidomide treatment. Hellstrom et al [39] studied the effect of lenalidomide on isolated differentiating erythroblasts from del 5q MDS patients and healthy controls. The addition of lenalidomide significantly inhibited the invitro proliferation of erythroblasts harboring del 5q while the proliferation of cells from normal controls and cells without 5q deletion was not affected. Gene expression profiling was performed at day 7 when a median of 97% cells in culture from MDS patients with del5q still possess del 5q, and thus any difference in gene expression deemed to be reflective of del 5q cells. There was altered gene expression in many genes, but a set of 4 genes was consistently upregulated (VSIG4, PPIC, TPBG and SPARC) by more than 2 fold in all samples. The upregulation of SPARC (Secreted Protein Acidic and Rich in Cysteine) after treatment with lenalidomide is particularly interesting given its location at 5q 31–32 and its role as a tumor suppressor with its anti-proliferative, anti adhesion, anti-angiogenic properties. The levels of activin -A increased 4 fold and analysis of global gene expression revealed significant deregulation of genes involved in extracellular matrix interactions, erythropoiesis relative to healthy control.

Another recent study compared gene expression profile of CD34 stem cells of 5q del MDS patients to healthy controls and MDS patients with normal karyotype using Affymetrix arrays. Approximately 40% of the probe sets showing reduced expression levels localized to the del 5q region. The commonly deleted region (CDR) region is thought to comprise of approximately 40 genes that are hypothesized to have a tumor suppressive role given the observation that deletion of the 5q region leads to clonal proliferation of myelodysplastic clone. Majority of the genes associated with CDR showed lower expression but several candidate genes (RBM22 and CSNK1A1, SPARC and RPS14) associated with CDR of the 5 q syndrome showed marked down regulation[40]. RBM22 is a highly conserved ribosomal protein, and the effects of downregulation may include deregulated apoptosis by its action on ALG-2(apoptosis linked gene). CSNK1A1 has recently been shown to be important in Hedgehog signaling that governs cell growth and a deregulation is observed in cancers. Downregulation of CSNK1A1 may contribute to MDS by altering the Hh signaling. RPS14 is related to the 40S subunit of the ribosome that is downregulated in Cd34 cells from MDS patients with del 5q[40]. Recent work shows that downregulation of RPS14 leads to defective erythropoiesis and increased apoptosis in erythroid progenitors [41].

Another candidate gene in the CDR region is Early growth response gene (EGR-1), that encodes a transcription factor involved in the regulation of cell proliferation and apoptosis[42]. The effect of lenalidomide treatment on expression of EGR-1 was studied in del 5q Burkitt's lymphoma and del 5q multiple myeloma cell line. It was observed that lenalidomide treatment did not influence the transcriptional activity of EGR-1 gene, but increased the nuclear export of EGR-1 in a dose dependent manner, especially in those with a single copy of EGR-1 gene. When the gene expression was blocked with an EGR1 siRNA, Burkitt's cells proliferated more than normal cells, supporting the tumor suppressor role of EGR-1 in Burkitt's cells. Thus, lenalidomide increases the nuclear transport of the pro apoptotic and tumor suppressor EGR-1, which could explain its cytotoxic effects on del 5q31 myelodysplastic clones.

In an effort to identify molecular markers of response to lenalidomide, Ebert et al [43] collected bone marrow aspirates of non 5 q del MDS patients before and after treatment with lenalidomide and studied the difference in gene expression between responders and non responders. Differential expression of the genes that needed for erythroid differentiation was noted in non responders than responders. In patients who responded to lenalidomide, they found that the bone marrow aspirates before treatment showed decreased expression of the set of the genes needed for erythroid differentiation. The thinking is that lenalidomide helps to overcome this differentiation block and hence the clinical response is seen in that subset of patients with decreased gene expression compared to the non responders. This was thought have potential predictability for benefit from lenalidomide therapy in non 5 q del patients.

A recent study by Wei et al [44] demonstrates that the haplodeficient enzymatic targets of lenalidomide within the CDR are dual specificity phosphatases, Cdc25C and PP2Acα. These phosphatases are coregulators of G2-M checkpoint in the cell cycle and thus, their inhibition by lenalidomide leads to G2 arrest and apoptosis. Since, most MDS patients including those with deletion 5q become refractory to Erythropoietin, the authors examined the molecular mechanisms by which lenalidomide may modulate this effect. They observed that the CD45 phosphatase is overactivated in MDS and may inhibit Epo receptor stimulated phosphorylation of stat5. Furthermore, they observed that lenalidomide is a Protein Tyrosine Phosphatase inhibitor of CD45 leading to reversal of CD45 induced inhibition of EPO-R/STAT5 signaling essential for hematopoiesis. The authors hypothesized that lenalidomide may thus be able to restore sensitivity to MDS by this mechanism. These concepts have led to clinical trial effort using lenalidomide in combination with erythropoietin in low grade MDS [45].

## Conclusion

**Figure 3:**
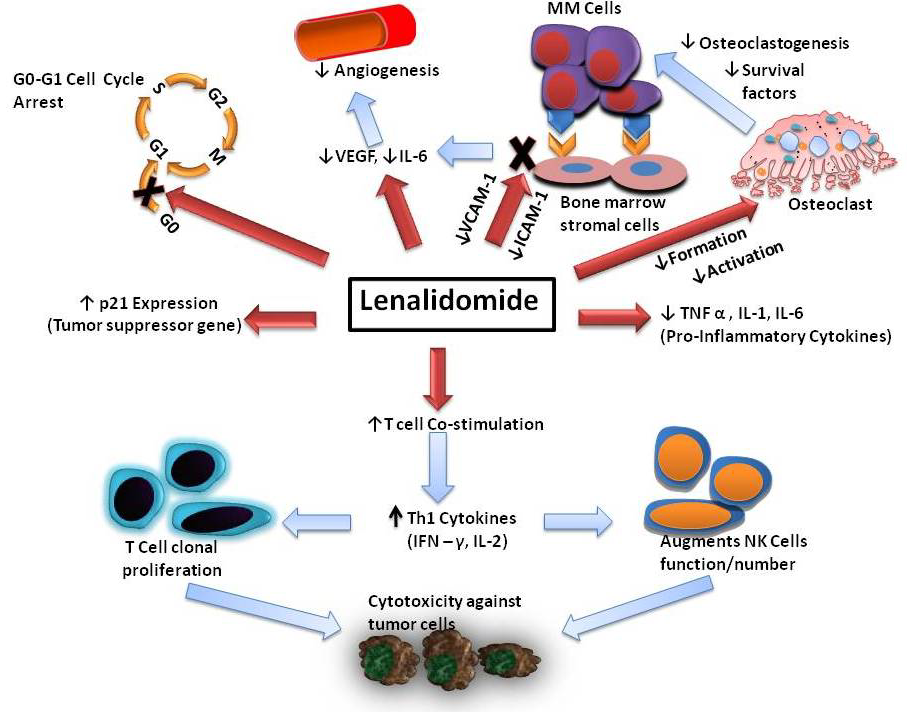
**Mechanism of action of lenalidomide**. Various mechanisms by which lenalidomide achieves clinical efficacy in hematological malignancies.

Lenalidomide has shown clinical efficacy in myelodysplasia [46-50], multiple myeloma [51-56], chronic lymphocytic leukemia [9,57-59], primary systemic amyloidosis [60,61], Non-Hodgkin's lymphoma [62], solid tumors [63-70], myelofibrosis with myeloid metaplasia [71] and Waldenstrom Macroglobulinemia [72]. It is also being increasedly used in combination with other chemotherapeutic agents. In relapsed multiple myeloma, it was combined with liposomal doxorubicin, vincristine and dexamethasone[53] as well as with adriamycin and dexamethasone[73]. Another combination being tested is lenalidomide with melphalan and dexamethsaone in treatment naïve myeloma[56]. A regimen combining lenalidomide with docetaxel and carboplatin has been tested in a phase 1 trial in advanced solid tumors[70]. Another very interesting combination is lenalidomide and rituximab in diseases such as NHL[74], CLL[9] and Waldenstrom Macroglobulinemia[72]. Preliminary results from some of these trials appear encouraging and final results are awaited. Even though various mechanisms have been proposed to explain its efficacy, as a single agent or in combination, in these conditions, the exact molecular and cellular targets of lenalidomide are not very well defined. It is possible that its efficacy is a result of its effects on the immune system, angiogenesis and signal transduction or a combination of all of these. Figure 3 summarizes the mechanism of action of lenalidomide as we know so far. Future studies will assess these mechanisms as well as direct actions on the malignant cells. These studies may uncover newer targets and lead to efforts to enhance the efficacy of this interesting new agent. These studies may also lead to development of newer IMiDs that may target specific mechanisms of action more potently, to further enhance their clinical activity and may provide an important biologic rationale to combine therapies with distinct, yet well defined site of action.

## Competing interests

The authors declare that they have no competing interests.

## Authors' contributions

Venumadhav Kotla and Swati Goel equally contributed to the extensive literature review and manuscript drafting. Sangeeta Nischal and Christoph Heuck participated in the literature review. Kumar Vivek participated in the literature review and designed the figures. Bhaskar Das provided the chemical names and the structures of the different compounds. Amit Verma conceived of the review, and participated in its design and coordination. All authors read and approved the final manuscript.
